# Effect of Dietary Methionine Deficiency Followed by a Re-Feeding Phase on the Hepatic Antioxidant Activities of Lambs

**DOI:** 10.3390/ani11010007

**Published:** 2020-12-23

**Authors:** Rong Liu, Qiyu Diao, Kai Cui

**Affiliations:** 1 Key Laboratory of Feed Biotechnology of the Ministry of Agriculture, Feed Research Institute, Chinese Academy of Agricultural Sciences, Beijing 100081, China; liurong_student@163.com (R.L.); diaoqiyu@caas.cn (Q.D.); 2 State Key Laboratory of Grassland Agro-Ecosystems, College of Pastoral Agriculture Science and Technology, Lanzhou University, Lanzhou 730020, China

**Keywords:** methionine, deficiency, antioxidants, lamb

## Abstract

**Simple Summary:**

Dietary methionine is closely related to oxidative stress metabolism. Lambs were treated with methionine restriction and subsequent methionine re-feeding. The relationship among dietary methionine pattern, methionine metabolism, and antioxidant reactions was studied by detecting methionine metabolite content and antioxidant expression in order to provide theoretical basis for amino acid nutrition and healthy feeding of lambs.

**Abstract:**

Our objective was to investigate the effect of methionine restriction and resuming supply on liver antioxidant response in lambs. The concentrations of methionine and its metabolites and the expression of the nuclear factor erythroid 2-related factor 2 (Nrf_2_), a redox sensitive factor, were detected after methionine restriction treatment for 50 days and methionine supply recovery for 29 days. The expression of glutathione (GSH) S-transferase (GST), superoxide dismutase (SOD), catalase (CAT), and glutathione peroxidase (GSH-Px) were characterized at the level of transcription and translation. Methionine restriction can directly change the content of methionine and its metabolites in plasma and liver, and affect the redox state of lambs by activating the Nrf_2_ signaling pathway. Liver tissue can adapt to oxidative environment by upregulating the expression of antioxidant enzymes such as GSH-Px and SOD. Moreover, it was found that there was a lag effect in the recovery of metabolism after methionine supplementation.

## 1. Introduction

Accumulating evidence suggests that nutrition exerts considerable effects on health, and one of the main ways to improve health is through dietary interventions [1,2]. In the past few years, a core aspect of anti-aging research has been anti-aging strategies, which include time-restricted feeding, calorie restriction, fasting mimicking diets, and short-/long-term fasting. These strategies are conducive to optimizing the nutritional balance, preventing or alleviating a variety of diseases, such as metabolic disturbance, cardiovascular diseases, and autoimmune diseases [3,4,5]. Methionine is an essential amino acid necessary for normal growth and development in mammals and assumes an important role in many physiological processes, such as metabolism, growth, and immunity [6,7]. Moreover, research continues to validate the critical importance that methionine restriction (MR) plays in decreasing body weight, reducing hepatic lipid levels and reactive oxygen species (ROS) production, increasing hepatic stress resistance, and preventing age-related diseases [8,9]. Even more striking, evidence from animal model studies shows that ROS production and oxidative damage can be reduced by dietary caloric or protein restriction, of which the essential reason is dietary methionine restriction. [10]. The addition of methionine or cysteine partially or completely eliminated the effect on the expression of glutathione (GSH) S-transferases (GSTs) mRNA in the liver of mice fed with protein–calorie malnutrition (PCM) or protein-free diet (PFD) [11,12].

The increased production of ROS is implicated in the altered redox balance, in which ROS attack cellular macromolecules and cause cellular lipid, protein, and DNA peroxidation damage, which is a common characteristic of aging [10]. Many studies have shown that MR has a positive effect on oxidative stress. MR can reduce mitochondrial ROS production and oxidative damage to mitochondria and systems [13,14,15]. Contrary to the beneficial effect of methionine restriction on oxidative stress, MR decreases the glutathione (GSH) level in several tissues [16,17]. As a metabolite of methionine, GSH is the most abundant non-protein sulfhydryl group in cells, which is a protective agent against oxidative stress [18]. Notably, the lack of GSH, the major non-enzymatic antioxidant, did not lead to an increase in oxidative damage to cells or tissues. In this regard, researchers tended to think that the decrease of the GSH level can be a key signal which can activate Nrf_2_ transcription and induce activation of a series of pathways related to antioxidation and then correct the decrease of GSH in liver and help to complete the metabolic adaptation during MR [19,20].

The family of glutathione S-transferases (GSTs) can protect cells from toxic chemical damage by catalyzing the combination of GST with electrophilic exogenous compounds [18,21]. The promoter region of the majority of the detoxifying and antioxidant enzyme genes including the α and μ forms of GST contain the antioxidant response element (ARE) which binds to Nrf_2_ and maintains the cellular redox homeostasis. The Nrf_2_–ARE signaling pathway is the most researched for candidates in antioxidant response [22]. Sulfur amino acids are closely related to the expression of GSTs in the liver and L-methionine and L-cysteine restriction can specifically upregulate the expression of glutathione S-transferase pi (GSTP) in primary hepatocytes of rats [11,23]. Lin et al. confirmed that MR activates Nrf_2_–ARE signaling pathway and drives Nrf_2_ binding to the enhancer I of GSTP (GPEI) in the GSTP promoter region, which induces the increase of phase II metabolizing enzyme GSTP [21]. In addition to GSTs, superoxide dismutase (SOD), glutathione peroxidase (GSH-Px), and catalase (CAT) also play an important role in scavenging ROS in mammals [18]. It is controversial whether antioxidant enzymes participate in the downregulation of oxidative stress during MR. Maddineni et al. found no changes in the activities of GSH reductase superoxide dismutase in livers of the rats that received MR feeding (80% dietary restriction of methionine (Met)). The finding indicates that oxidative stress is reduced by MR feeding in rats, but this effect cannot be explained by changes in the activity of antioxidant enzymes [16]. Meanwhile, part of the research suggests that the reduction of oxidative damage caused by MR is due to the decrease of ROS production but not the expression of antioxidant enzymes [14,16]. Tamanna’s report shows that the two “redox buffering” thiol systems compensate each other in MR. The increase of thioredoxin antioxidant system compensates for the decrease of the glutathione system [20].

Extensive research confirmed that reduction in the levels of oxidative stress may be a contributing mechanistic factor for the beneficial effects of MR [6,16,20]. However, it is controversial whether antioxidant enzymes are involved in the reduction of oxidative stress induced by MR [16,20]. In order to elucidate the underlying mechanism of MR alleviating the oxidative stress, we conducted this research by dietary intervention of methionine deficiency followed by a re-feeding phase with lambs. This dietary intervention mode helps to more accurately explore the mechanism of MR-mediated alleviation of the oxidative stress and provide a basic reference for designing personalized nutritional diet patterns.

## 2. Materials and Methods

The trial was conducted at Linqing Runlin Animal Husbandry, Shandong, China (36.680 N, 115.720 E). The Chinese Academy of Agricultural Sciences Animal Ethics Committee approved the experimental protocol, and all the methods used in this experiment were in accordance with humane animal care and handling procedures (AEC-CAAS-2017-02).

### 2.1. Animals and Diets

Twenty-four male Hu lambs (weaned at 7 days of age) with an initial body weight of 4.93 ± 0.20 kg were randomly assigned to two groups with 12 lambs per treatment and 1 lamb per replicate. The trial lasted 76 days and consisted of a methionine restriction period (8 to 56 days of age) and methionine recovery period (57 to 84 days of age). During the methionine restriction period, the lambs in the control (CON) group were supplemented with a milk replacer and a starter containing 0.91% and 0.60% Met, while the lambs in the MR group were fed a milk replacer and a starter containing 0.21% and 0.20% Met (dry matter (DM) basis), respectively. During the methionine recovery period, all lambs were weaned off the milk replacer and fed with the same starter containing 0.60% Met. The starter was made into pellets with a diameter of 6 mm and a length of 4–6 cm. During the experimental period, all lambs were housed in indoor pens, and the water was available ad libitum. The milk replacer and starter were offered and refusals were recorded every day to calculate the methionine intake. The composition of the milk replacer and the ingredients and composition of the starter are presented in Table 1.

### 2.2. Methionine Intake

0.7% and 0.4% DL-Met was added into the diets to formulate the baseline milk replacer and starter containing 0.91% and 0.6% Met, respectively. The added DL-Met was deducted from the baseline diets to formulate Met-deficient milk replacer and starter containing 0.21% and 0.2% Met, respectively, which were the components of the baseline diet. The feed was offered and refusals were recorded daily to calculate methionine intake.

### 2.3. Serum Profiles

Blood samples were collected from 6 lambs of each group before morning feeding by jugular vein puncture into 10 mL vacutainer tubes at the age of 56 and 84 days, respectively. Blood samples were centrifuged for 15 min at 3400 rcf and the serums were transferred into 1.5 mL plastic tubes and stored at −20 °C. The activity of catalase (CAT), superoxide dismutase (SOD), glutathione peroxidase (GSH-Px), and glutathione S-transferases (GSTs) was analyzed with commercial kits from the Nanjing Jiancheng Bioengineering Institute (Jiangsu, China) according to the manufacturer’s protocols.

### 2.4. Determination of Methionine and Metabolites

A double-antibody sandwich ELISA was selected for the detection of the serum and hepatic content of methionine and its metabolites. Methionine, S-adenosyl methionine, S-adenosyl homocysteine, and homocysteine were detected with commercial kits according to the manufacturer’s protocols. All kits were obtained from Zhenke Biotechnology Co. Ltd (Shanghai, China).

### 2.5. Total RNA Extraction and Quantitative RT-PCR

Total RNA of liver was isolated with the Trizol reagent according to the manufacturer’s instructions (Invitrogen, USA). The first-strand complementary DNA (cDNA) was synthesized from 1 μg of the purified total RNA using Fast Quant RT Kit (TianGen, China). An SYBR green-based qRT-PCR kit was employed and the expression analysis was evaluated using an iQ5 real-time PCR detection system (Bio-Rad; Hercules, CA, USA) relative to the expression of β-actin, which was used as housekeeping gene (internal references). The relative expression levels were assessed in triplicate and calculated using the 2^−ΔΔCT^ method (Livak and Schmittgen, 2001). The primers used in this study were synthesized by TsingKe Co. Ltd. (Beijing, China) and are listed in Table 2.

### 2.6. Western Blot Analysis

The liver tissue was homogenized in the ice-cold Radio-Immunoprecipitation Assay (RIPA) buffer (Beyotime, China) for 30 min and then centrifuged (10,000× *g*, 20 min, 4 °C). The supernatant was collected and the protein concentration was measured by the bicinchoninic acid (BCA) method with bovine serum albumin as the standard. Proteins (50 ng) were separated by SDS-PAGE and subsequently transferred to nitrocellulose filter (NC) membranes. Membranes were blocked with 5% non-fat dry milk in the incubation buffer and incubated with the rabbit anti-catalase antibody (1:1000 dilution), rabbit anti-GPX1 antibody (1:1000 dilution), rabbit anti-GSTA3 antibody (1:1000 dilution), and rabbit anti-SOD1 antibody (1:1000 dilution), respectively. Then, the membranes were washed in Tris-buffered saline Tween-20 (TBST). A horseradish peroxidase-linked antibody (goat anti-rabbit IgG/HRP (horseradish peroxidase) antibody, 1:5000 dilution) was employed as the secondary antibody. All antibodies were obtained from Biosynthesis Biotechnology Co. Ltd (Beijing, China). Image Studio software was used to analyze the bands, and the expression ratios were normalized to β-actin.

### 2.7. Statistical Analyses

Statistical analysis was performed with the SAS statistical software (version 9.2, SAS Institute Inc., Cary, NC, USA) [24]. In the statistical analysis of the expression of antioxidant mRNA and proteins in the liver of lambs, individual lambs were taken as experimental units, and the data were analyzed by the independent sample *t*-test. The other data were analyzed using paired *t*-tests with individual lambs as the experimental units, and the pairs in a paired *t*-test were the two members of each set of twins. Results with *p* < 0.05 were considered significant.

## 3. Results

### 3.1. Methionine Intake

During the methionine restriction period, the intake of milk replacer and starter showed no difference between the two groups (*p* > 0.05). The methionine intake of lambs in the MR group was lower than that of the CON group (*p* < 0.05). During the follow-up Met replenishment period, there were no differences in the starter feed and Met intake between groups (*p* > 0.05) (Table 3).

### 3.2. Methionine Metabolite Content

Compared to the CON group, methionine restriction decreased the serum content of methionine and S-adenosyl methionine (*p* < 0.05) and no difference was detected in the serum content of S-adenosyl homocysteine and homocysteine (*p* > 0.05). After a 28-day refeeding period, the serum content of methionine and S-adenosyl methionine was still lower in the MR group than in the CON group (*p* < 0.05) (Table 4). During the methionine restriction period, the liver methionine content of lambs in the MR group was higher than that in the CON group (*p* < 0.05). The content of S-adenosyl methionine, S-adenosyl homocysteine, and homocysteine showed no difference between the two groups (*p* > 0.05). After a 28-day refeeding period, the content of methionine, S-adenosyl methionine, and S-adenosyl homocysteine was lower than that in the CON group (*p* < 0.05). No difference was observed in the content of homocysteine between the two groups (*p* > 0.05) (Table 5).

### 3.3. Serum Antioxidant Enzyme Activities

Compared to the CON group, methionine restriction decreased the activity of SOD during the methionine restriction period (*p* < 0.05). No difference was observed in the activity of GST, GSH-Px, and CAT between the two groups (*p* > 0.05) (Table 6). Serum SOD activity of the MR group was fully normalized with the replenishment of methionine. During the follow-up Met re-feeding period, the activity of GSH-Px of the MR group was decreased (*p* < 0.05), while no difference was detected in the GST and CAT activities (*p* > 0.05).

### 3.4. Quantitative Real-Time PCR Analysis

**Figure 1:**
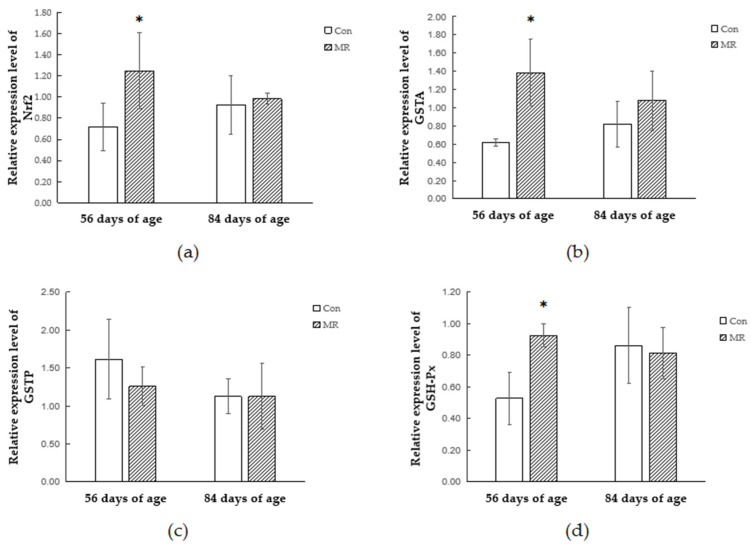

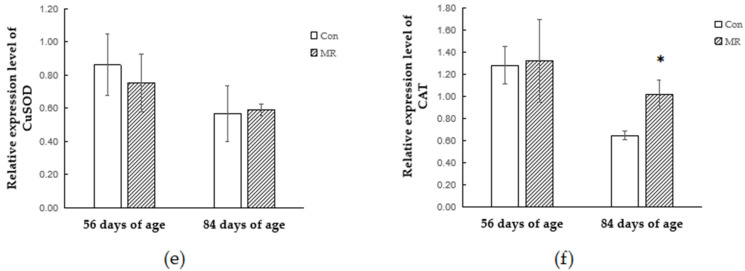
Effects of dietary methionine deficiency and supplementation on mRNA expression of antioxidants in the liver of lambs. (**a**) Expression of Nrf_2_ mRNA. (**b**) Expression of GSTA mRNA. (**c**) Expression of GSTP mRNA. (**d**) Expression of GSH-Px mRNA. (**e**) Expression of CuSOD mRNA. (**f**) Expression of CAT mRNA. The results are expressed as means ± SD, *n* = 4, and the bars represent means of triplicate determinations, * *p* < 0.05.

The mRNA transcription levels of antioxidant-related enzymes and genes were assessed by qRT-PCR (Figure 1). At the age of 56 days, the mRNA expression of Nrf_2_, GSTA, and GSH-Px in the MR group was higher than that in the CON group (*p* < 0.05). However, no difference in the mRNA expression of GSTP, CuSOD, and CAT was detected between the CON group and the MR group (*p* > 0.05). After a 28-day re-feeding period, the mRNA expression of CAT in the MR group was higher than that in the CON group (*p* < 0.05). The replenishment of methionine affected the mRNA expression of Nrf_2_, GSTA, GSH-Px, GSTP, and CuSOD (*p* > 0.05) (Figure 1).

### 3.5. Protein Expression

**Figure 2:**
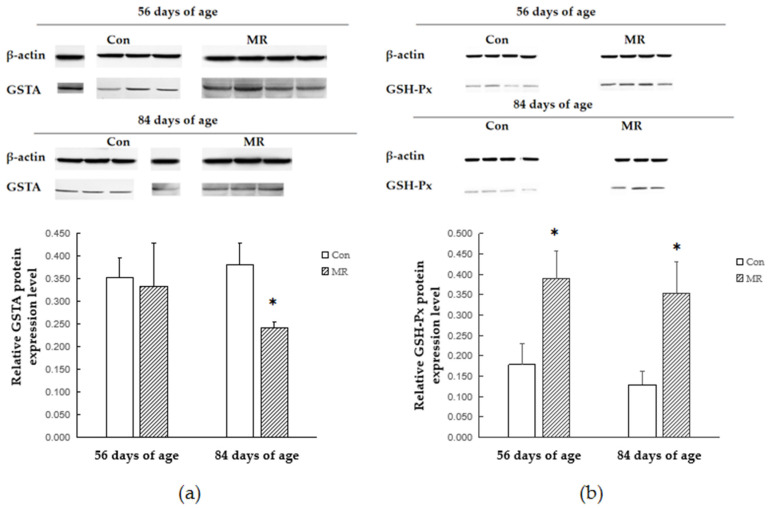

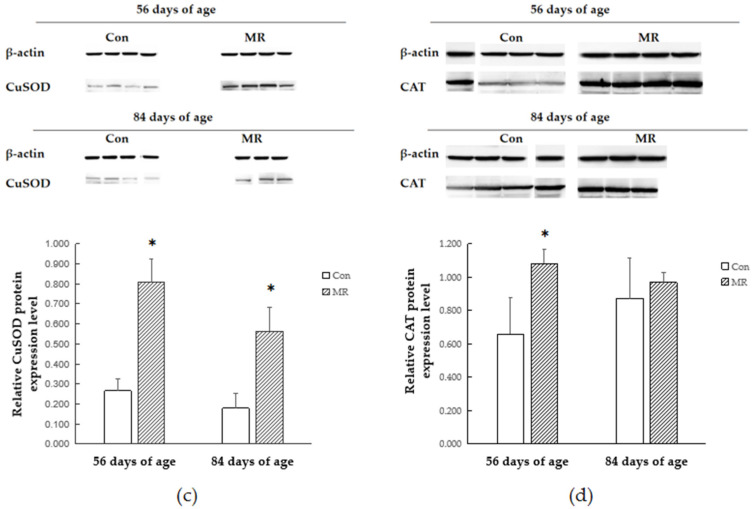
Effects of dietary methionine deficiency and supplementation on protein expression of antioxidants in the liver of lambs. (**a**) Relative expression of the GSTA protein. (**b**) Relative expression of the GSH-Px protein. (**c**) Relative expression of the CuSOD protein. (**d**) Relative expression of the CAT protein. The results are expressed as means ± SD, *n* = 4, and the bars represent means of triplicate determinations, * *p* < 0.05. One sample was missing in the MR group at 84 days of age.

The relative protein expression levels are shown in Figure 2. The gray value method and Lab Works 4.6 software were used for the relative expression analysis of related proteins and β-actin, which was designated as the reference protein in each sample. At 56 days of age, the protein levels of CuSOD and GSH-Px in the MR group were higher than those in the CON group (*p* < 0.05), while the protein levels of CAT exhibited a rising trend. There was no difference in the protein expression level of GSTA between the two groups (*p* > 0.05). At 84 days of age, the protein levels of CuSOD and GSH-Px in the MR group were higher than those in the CON group (*p* < 0.05). The protein level of GSTA was lower than that in the CON group (*p* < 0.05; Figure 2a). No difference in the protein expression level of CAT was observed between the two groups (*p* > 0.05) (Figure 2).

## 4. Discussion

Methionine is an essential amino acid and must be obtained from the diet to sustain life. Researchers have demonstrated the critical importance of limiting methionine in the diet of animals or in cell culture media, which could provides metabolic benefits such as decreasing adiposity [25,26,27], increasing insulin sensitivity [28,29], decreasing inflammation [13,16,30] and oxidative stress [31], and extending lifespan [32].

It is reported that 80% methionine restriction affected oxidative stress and glutathione-related redox pathways in rats [16,19,33]. In this study, the methionine intake of lambs in the MR group and the CON group was ~0.47 g DM/day and 1.75 g DM/day, respectively. Compared to the CON group, the restriction level of the MR group was 73.14%, which is consistent with previous studies. We found that methionine restriction significantly decreased the serum content of methionine and S-adenosyl methionine, and the methionine replenishment of 28 days was not enough to bring normal methionine levels back. Liver is an important site for methionine metabolism. Therefore, the methionine and metabolite content of liver tissues was also determined in this study. Contrary to the serum findings, the methionine restriction increased the liver methionine content. We also found that methionine and its metabolites such as S-adenosyl methionine and S-adenosyl homocysteine were significantly lower than in the CON group after the 28-day re-feeding phase, which was consistent with the study of Atlantic salmon where methionine limitation reduced free methionine concentration in the plasma and muscles, while methionine concentration in the liver was not affected [34]. Since MR decreases serum methionine concentrations, it was speculated that hepatic metabolism could act to compensate for the reduction of methionine intake. The lagging of hepatic metabolism might be the reason of lower methionine concentration during the methionine replenishment period.

The activity of antioxidant enzymes in the serum reflected the redox states of lambs. In the present study, methionine restriction decreased the SOD activity and the GSH-Px activity was significantly lower during the methionine replenishment period. It can be inferred that methionine restriction will have a negative effect on the antioxidant capacity of lambs and there will be a continuous effect after the dietary methionine is adequate. However, a study of weaned piglets showed that the activity of serum SOD in the MR group was higher than in control groups, and the total antioxidant capacity of the MR group was lower [35]. Given that this study confirmed that the activity of antioxidant enzymes differs in different tissues, such as liver, kidney, blood, muscle, and adipose tissue, we may be certain that methionine restriction can directly lead to changes in tissue oxidative status.

The Nrf_2_–ARE pathway is an indicator and modulator of oxidative stress and activation of this pathway protects cells from oxidative stress-induced cell death [36]. In this study, methionine restriction increased the mRNA expression of Nrf_2_ and GSTA genes in lambs, but no difference was found in the expression of GSTP. The expression of Nrf_2_ and GSTA returned to its normal level after dietary methionine became adequate. Lin (2012) reported that MR can activate the extracellular signal-regulated kinase (ERK)–Nrf_2_ signaling pathway and drives Nrf_2_ binding to the GSTP promoter region and upregulates transcription of target genes [21]. A similar result was found in mice which were fed a protein-free diet: levels of the GSTA and the GSTP in the liver were increased, whereas normal contents were preserved in the rats fed a protein-free diet replenished with L-methionine [11]. Consistent with our research, these findings suggest that the expression of certain GST genes can be modulated depending on the cellular L-methionine status and Nrf_2_ plays the key role in the process.

In order to study whether and how antioxidant enzymes participate in the antioxidant response during methionine restriction, we detected the alteration of the antioxidant enzyme expressing style at the gene transcription and translation levels. The protein encoded by GPX1 belongs to the glutathione peroxidase family, members of which catalyze the reduction of organic hydroperoxides and hydrogen peroxide (H_2_O_2_) by glutathione and thereby protect cells against oxidative damage [37]. Methionine restriction increased the expression of GSH-Px at both the transcriptional and translational levels, which might be the reason of reduction in free radicals. The SOD protein binds copper and zinc ions and acts as a homodimer to convert naturally occurring but harmful superoxide radicals to molecular oxygen and hydrogen peroxide [38]. This study showed that methionine restriction increased the expression level of SOD and the protein expression of methionine restriction group was still higher than that in the control group after methionine supply was restored. The increase of the antioxidant enzyme protein level may be due to the increase of the balanced body oxidation tendency, which is caused by the decrease of methionine metabolite GSH. However, the transcriptional changes of antioxidant enzymes were not consistent with their respective protein changes under methionine restriction. Previous research confirmed that the increase of antioxidant enzyme mRNA during oxidative stress is not always associated with the increase of enzyme activity or protein content in mammalian cells and tissues [39,40,41]. It is closely related to the free radical scavenging process and redox state of a mammal.

## 5. Conclusions

This study demonstrated that methionine restriction affected the redox state of lambs. Upregulation of the GSTA gene expression appears to be related to the deficiency of the GSH precursor, which in turn activates the Nrf_2_ signaling pathway. During the methionine restriction period, cells accommodate themselves to the deficient methionine conditions through the upregulation of antioxidant enzymes such as GSH-Px and SOD in order to maintain the redox state. The cellular homeostasis did not return to normal levels, which might have resulted from the lagging hepatic methionine metabolism during the methionine replenishment period.

## Figures and Tables

**Table 1:** Ingredients and composition of the baseline milk replacer and the baseline starter (dry matter basis) ^A^.

Items	Baseline Milk Replacer ^B^	Baseline Starter
Ingredients (% of DM)		Content
Corn meal		65.07
Wheat bran		15.00
Soybean meal		5.58
Limestone		1.90
Fat powder		2.00
Dicalcium phosphate		1.51
NaCl		0.79
DL-methionine		0.40
Compound amino acids ^C^		6.75
Premix ^D^		1.00
Total		100.00
Nutritional composition ^E^		
DM (%, air-dried basis)	95.69	88.20
CP (%)	21.66	16.16
EE (%)	6.44	4.68
Ash (%)	5.88	9.38
Ca (%)	1.02	1.23
TP (%)	0.51	0.54
NDF (%)	2.63	28.32
ME (MJ/kg)	15.10	10.75
Met (%)	0.91	0.60
Try (%)	0.29	0.18
Lys (%)	2.77	1.01
Thr (%)	1.17	0.60

^A^ 0.7% and 0.4% DL-Met were added into the diets to formulate the baseline milk replacer and starter containing 0.91% and 0.6% Met, respectively. The added DL-Met was deducted from the baseline diets to formulate Met-deficient milk replacer and starter containing 0.21% and 0.2% Met, respectively, which were the components of the baseline diet. Other than those made to the Met content, no other changes were made to the CON or methionine-deficient (MD) diets. ^B^ Due to the patent application status, the composition of the milk replacer was not given. The milk replacer was provided by the Beijing Precision Animal Nutrition Research Center. ^C^ The compound amino acid is composed of lysine, tryptophan, threonine, valine, histidine, and other amino acids. ^D^ One kg of the premix contained the following: Fe: 22.10 g, Mn: 9.82 g, Cu: 2.00 g, Zn: 12.00 g, Se: 20 mg, I: 200 mg, Co: 50 mg, vitamin A: 1,300,000 IU, vitamin D_3_: 350,000 IU, vitamin E: 4000 IU. ^E^ All items are on the DM basis except DM%, which is a % of the “as fed” diet. All the data represent measured values except ME, which was calculated based on the Tables of Feed Composition and Nutritive Values in China 2012. DM: dry matter; CP: crude protein; EE: ether extract; TP: total phosphorus; NDF: neutral detergent fiber; ME: metabolizable energy; Met: methionine; Try: tyrosine; Lys: lysine; Thr: threonine.

**Table 2:** Sequences of the primer pairs used in the quantitative real-time PCR assays.

Gene	Forward (5′–3′)	Reverse (5′–3′)	GenBank Accession of mRNA
β-actin	CTCACGGAGCGTGGCTACA	GCCATCTCCTGCTCGAAGTC	NM_001009784.3
Nrf_2_	TCTGCCAACTACTCCCAGGT	AGGAGCATTGAAGACTGGGC	XM_012132956.3
GSTA	GACCAGAGCCATTCTCAACTAC	CTTTTCTCGGATTAGGGTCAG	XM_027959005.1
GSTP	ACGGAGACCTCACCCTTTAC	TTTGTCCTCCTCACGCTTG	XM_027959471.1
CuSOD	AGGCAAAGGGAGATAAAGTCGTCG	TGCACTGGTACAGCCTTGTGTATTG	NM_001145185.2
GSH-Px	CCAAGAACGAGGAGATCCTG	ACTTAGGGTCGGTCATGAGAG	XM_004018462.4
CAT	CCTATCCTGACACTCACCGC	CATCGCTGGCACTGTTGAAG	XM_012096208.3

Nrf_2_: nuclear factor E2-related factor 2; GSTA: glutathione S-transferase alpha; GSTP: glutathione S-transferase pi; CuSOD: copper-containing superoxide dismutase; GSH-Px: glutathione peroxidase; CAT: catalase.

**Table 3:** Effect of dietary methionine deficiency followed by replenishment on growth performance.

Items	CON	MR	SEM	*p*-Value
Days 8 to 56				
Milk replacer intake (g DM/d)	131.41	131.19	-	-
Starter intake (g DM/d) ^A^	91.64	97.45	2.90	0.071
Methionine intake (g DM/d) ^A^	1.75 ^a^	0.47 ^b^	0.02	<0.001
Days 57 to 84				
Starter intake (g DM/d) ^B^	517.32	532.77	8.70	0.136
Methionine intake (g DM/d) ^B^	3.10	3.19	0.05	0.140

CON: control diet, including the baseline milk replacer and starter with 0.91% and 0.60% Met, respectively. MD: methionine-deficient diet, including the milk replacer and starter with 0.21% and 0.20% Met, respectively. ^A^ *n* = 3/group, 3 pens of 4 lambs per group. ^B^ *n* = 3/group, 3 pens of 2 lambs per group, ^a,b^ values in the same row are different at *p* < 0.05.

**Table 4:** Effect of dietary methionine deficiency followed by replenishment on methionine cycle metabolite contents in the serum of lambs.

Item	Groups		SEM	*p*-Value
	CON	MR		
56 days of age				
Met (ng/mL)	264.17 ^a^	199.05 ^b^	15.10	0.0231
SAM (ng/mL)	11.99 ^a^	9.62 ^b^	0.64	0.0341
SAH (ng/mL)	9.16	8.24	0.66	0.2993
Hcy (ng/mL)	7.20	5.38	0.88	0.1736
84 days of age				
Met (ng/mL)	259.92 ^a^	208.92 ^b^	18.91	0.0433
SAM (ng/mL)	11.13 ^a^	9.44 ^b^	0.49	0.0420
SAH (ng/mL)	11.75	10.61	0.47	0.0930
Hcy (ng/mL)	7.35	6.93	0.32	0.3223

Met: methionine; SAM: S-adenosyl methionine; SAH: S-adenosyl homocysteine; Hcy: homocysteine. ^a,b^ values in the same row are different at *p* < 0.05.

**Table 5:** Effect of dietary methionine deficiency followed by replenishment on methionine cycle metabolite contents in the liver of lambs.

Item	Groups		SEM	*p*-Value
	CON	MR		
56 days of age				
Met (ng/mL)	236.37 ^b^	252.23 ^a^	3.57	0.047
SAM (ng/mL)	3389.93	3455.49	192.03	0.750
SAH (ng/mL)	3768.22	3419.33	365.67	0.384
Hcy (ng/mL)	766.91	784.97	155.66	0.913
84 days of age				
Met (ng/mL)	284.33 ^a^	175.74 ^b^	13.39	0.015
SAM (ng/mL)	4053.18 ^a^	2865.50 ^b^	236.35	0.007
SAH (ng/mL)	4639.28 ^a^	3603.02 ^b^	305.98	0.020
Hcy (ng/mL)	849.87	841.30	169.91	0.962

Met: methionine; SAM: S-adenosyl methionine; SAH: S-adenosyl homocysteine; Hcy: homocysteine. ^a,b^ values in the same row are different at *p* < 0.05.

**Table 6:** Effect of dietary methionine deficiency followed by replenishment on serum antioxidant indexes in lambs.

Items	Groups		SEM	*p*-Value
	CON	MR		
56 days of age				
GST (U/mL)	271.61	276.3	10.13	0.6631
SOD (U/mL)	362.02 ^a^	284.95 ^b^	17.4	0.0068
GSH-Px (U/mL)	21.87	20.36	1.14	0.2432
CAT (U/mL)	49.91	56.01	6.78	0.4097
84 days of age				
GST (U/mL)	279.69	260.42	16.53	0.2964
SOD (U/mL)	365.08	338.53	31.15	0.433
GSH-Px (U/mL)	25.42 ^a^	20.11 ^b^	1.4	0.0128
CAT (U/mL)	56.99	54.8	7.76	0.7896

GST: glutathione S-transferase; SOD: superoxide dismutase; GSH-Px: glutathione peroxidase; CAT: catalase. ^a,b^ values in the same row are different at *p* < 0.05.

## Data Availability

Data is contained within the article or supplementary material. The data presented in this study are available in [this paper].
